# Gaps between Activities of Daily Living Performance and Capacity in People with Mild Dementia

**DOI:** 10.3390/ijerph192315949

**Published:** 2022-11-30

**Authors:** Kosuke Nakanishi, Takayoshi Yamaga, Masanao Ikeya

**Affiliations:** Department of Occupational Therapy, Faculty of Medical Science, Nagoya Women’s University, 3-40 Shioji-cho, Mizuho-ku, Nagoya 467-8610, Japan

**Keywords:** dementia, IADL, gap, performance, capacity

## Abstract

Persons with mild dementia can effectively maintain and improve their quality of life (QOL) by regularly performing their daily activities. However, research on activities of daily living (ADL) in this population often fails to distinguish between ADL performance and ADL capability, that is, actual independence in daily lives and potential independence in an ideal setting. This study aimed to identify the potential gaps between ADL performance and capability in individuals with mild dementia. A total of 137 community-dwelling older adults (aged ≥ 65 years) who had been diagnosed with dementia and assessed as 0.5 and 1 on a global clinical dementia rating (CDR). Participants were evaluated for basic ADL (BADL) and instrumental ADL (IADL) using the Hyogo Activities of Daily Living Scale (HADLS). Around 35 individuals who met the inclusion criteria were enrolled in the study. BADL performance and BADL capacity were not significantly different (*p* = 0.128); however, participants rated IADL capacity significantly higher than IADL performance (*p* < 0.01). Gaps between performance and capability were observed for IADL but not for BADL. This study distinguishes between ADL performance and capability in individuals with mild dementia and is the first to identify the IADL-specific gap between these two components; pertinent targeted interventions are vital in closing this gap. Environmental adjustments are important to improve QOL of persons with mild dementia.

## 1. Introduction

Dementia has become a global challenge, with more than 55 million people worldwide currently suffering from dementia, a figure that is expected to increase to 139 million by 2050 [1]. Furthermore, it has physical, psychological, social, and economic consequences not only for the individual, but also for caregivers, family members, and society as a whole [1].

Dementia is a disease characterized by the chronic progression of core symptoms such as memory impairment and disorientation [1]. Memory impairment, executive dysfunction, poor judgment, and diminished comprehension are the most common symptoms associated with the early stages dementia for which no cure is currently available [1]. Therefore, core symptom improvement is not the primary outcome of nonpharmacological therapies for the disease. Quality of life (QOL) has attracted attention in recent years as an essential outcome when evaluating interventions for dementia [2]. This concept has been linked to activities of daily living (ADL) performance status in people with dementia [3,4,5], leading some to consider evidence-based ADL interventions as a necessary component of rehabilitation for this population [6].

ADL consists of basic ADL (BADL), such as self-care, and instrumental ADL (IADL), for example, preparing meals, cleaning, laundry [7]. In terms of ADL function, subtle declines in BADL from the mild cognitive impairment (MCI) stage should also be considered, but they tend to be generally maintained in the early stages of dementia, and impairment first appears in IADLs, which consist of complex processes requiring higher cognitive functions [8,9]; instead, impairments first manifest in IADL, consisting of complex processes requiring higher-order cognitive functioning [10,11]. In addition, ADL can be divided into ADL that are performed daily (ADL performance) and ADL that are capable of being performed but are not performed (ADL capacity). Performance ADL are “what the subject actually performs in daily life”, while capacity ADL are “what the subject can engage in using appropriate human support and environmental factors such as the use of welfare aids, as well as the subject’s fullest potential”. In cases where ADL capacity are not being demonstrated, in preparing meals, there are cases where even though they can put the dishes on the table by themselves (ADL capacity), they have others do it for them (ADL performance).

Discrepancies between ADL performance and ADL capacity, that is, between the actual performance of ADL in the elderly in everyday living environment and what they can achieve in a controlled environment with the necessary tools (physical environmental factors such as products, technology, etc.) and human factors such as appropriate support, relationships and attitudes, have long been noted in ADL assessments of the elderly [12]. On the other hand, discrepancy between these ADL, i.e., as the existence of a “gap” between ADL performance and capacity has not yet been explicitly demonstrated in people with mild dementia, contemporary ADL interventions in this population tend to be selected and adjusted through trial and error. Consequently, the ADL gap can reduce the potential capacity.

It is essential to distinguish between ADL performance and capacity as both can provide pertinent assessment information by demanding ADL-targeted interventions to help persons with dementia achieve full ADL potential in their daily routines [12,13].

This study’s objective was to determine whether a gap between ADL performance and ADL capacity exists among individuals with mild dementia to develop ADL intervention guidelines for this population.

## 2. Materials and Methods

### 2.1. Research Design

This cross-sectional study aimed to determine the status of ADL implementation in community-dwelling individuals with mild dementia.

### 2.2. Participants

The authors invited more than 100-day care centers in four regions of Japan to participate by mail. 26-day care centers expressing interest in the study were visited by the authors to convey additional detailed information about the study. A day care center is a place where people living at home in the community have the opportunity to engage in activities and socialize during the day. The participants of this study are those who use those day care centers. In order to carefully assess the subject’s usual ADL performance, those who had been using the day care center for at least one month were included. Candidates were recommended by the day care center’s manager. A total of 137 community-dwelling older adults (age ≥ 65 years) who had been diagnosed with dementia using the long-term care insurance system of Japan were evaluated. They were enrolled if they had a global clinical dementia rating (CDR) of either 1 or 0.5 and were mobile indoors without the use of a wheelchair. Candidates were excluded if they lived alone, had a severe neurological or musculoskeletal disorder that interfered with physical activity, severe problems with cardiopulmonary or circulatory function, severe vision or hearing loss, or a history of aggressive or violent behavior noted by the care staff. When diagnosed with dementia, participants did not undergo formal cognitive screening, diagnostic imaging, or pathological testing to comprehensively explore their diagnosis and disease stage owing to personal financial constraints.

The study subjects are people with dementia. Caregivers are in a position to evaluate them along with occupational therapists. Caregivers included in the study were those working at the same day care center as the participants, with no restriction on the caregivers’ profession, who directly took part in the care of the participants, and were most aware of information about the participants, including physical and psychological symptoms over the past month. Candidates were asked to provide written informed consent; when they were unable to provide it, their substitutes were asked to sign the consent form on their behalf.

### 2.3. Assessment Method

Participants were examined using the Hyogo Activities of Daily Living Scale (HADLS) [14]. The HADLS covers BADL and IADL. It is an integrated ADL assessment instrument covering 18 items of BADL and IADL, specifically for people with dementia. BADL covers toileting, feeding, dressing, grooming, washing, brushing teeth (or cleaning dentures), and bathing whereas IADL covers range of mobility, telephoning, shopping, preparing meals, cleaning, futon (bed) management, cleaning up after meals, laundry, handling (open) flames, handling switches, and money management. Higher HADLS values signify lower levels of independence. Participants’ ADL performance, that is, their real-world performance of the activity in question, was assessed by an occupational therapist and caregiver on their care team most familiar with their normal living conditions. In addition, they assessed ADL capacity in a controlled environment. In short, in the day care center, the occupational therapists set up a simulated environment that they considered necessary when assessing capacity ADLs, such as a microwave oven for meal preparation, with the necessary tools (physical environmental factors such as product, technology, etc.) and degree of support (human factors such as support, relationships, attitudes, etc.). The occupational therapist and caregiver then evaluated how much capacity the participants could demonstrate in that environment.

Cognitive function was evaluated using the Mini-Mental State Examination (MMSE) and was conducted through face-to-face interviews with patients [15]. CDR evaluation is a test that indicates the severity of dementia on a 6-point scale from 0 to 5, ranging from mild to severe, based on information obtained from interviews and caregivers [16]. In this study, CDR 0.5 to 1 was defined as mild dementia based on the results of interviews with participants and information from those who were aware of participants’ daily lives. Necessary information was obtained from the participants’ medical records.

### 2.4. Data Analysis

Data were analyzed using IBM SPSS Statistics 26.0 (IBM Inc., Armonk, NY, USA) for Macintosh. All variables were confirmed for a non-normal distribution prior to analysis. The Wilcoxon signed-rank test is used to test for significant differences between two conditions of an independent variable data is collected from the same participants.

In this study, as intra-individual changes due to environmental adjustments in ADLs, comparisons of median the performance and capacity scores for BADLs and median the performance and capacity scores for IADLs were analyzed using the Wilcoxon signed-rank test, with *p* < 0.05 being considered statistically significant.

### 2.5. Ethical Considerations

The study was conducted in accordance with the Declaration of Helsinki, and the protocol was approved by the Research Ethics Review Committee of the Health Science University (accession no. 1). The aims and methods of the study were explained to the participants and their substitutes. All participants gave their informed consent for inclusion before they participated in the study.

## 3. Results

### 3.1. Participant Demographics

Of the 137 patients, 35 individuals who met the criteria were enrolled (male: 8, female: 27; mean age: 86.5 ± 6.4). A total of 12 and 23 participants were rated as having CDR 0.5 and 1, respectively. The median (IQR) MMSE was 21.0 (19.0–22.0).

### 3.2. Assessment Results

On the HADLS, participants’ median BADL performance and BADL capacity were 4.5 (2.9–10.2) and 4.5 (2.8–10.0), respectively. In comparison, the median IADL performance and IADL capacity were 31.9 (24.9–37.5) and 25.3 (21.4–28.8), respectively.

**Figure 1:**
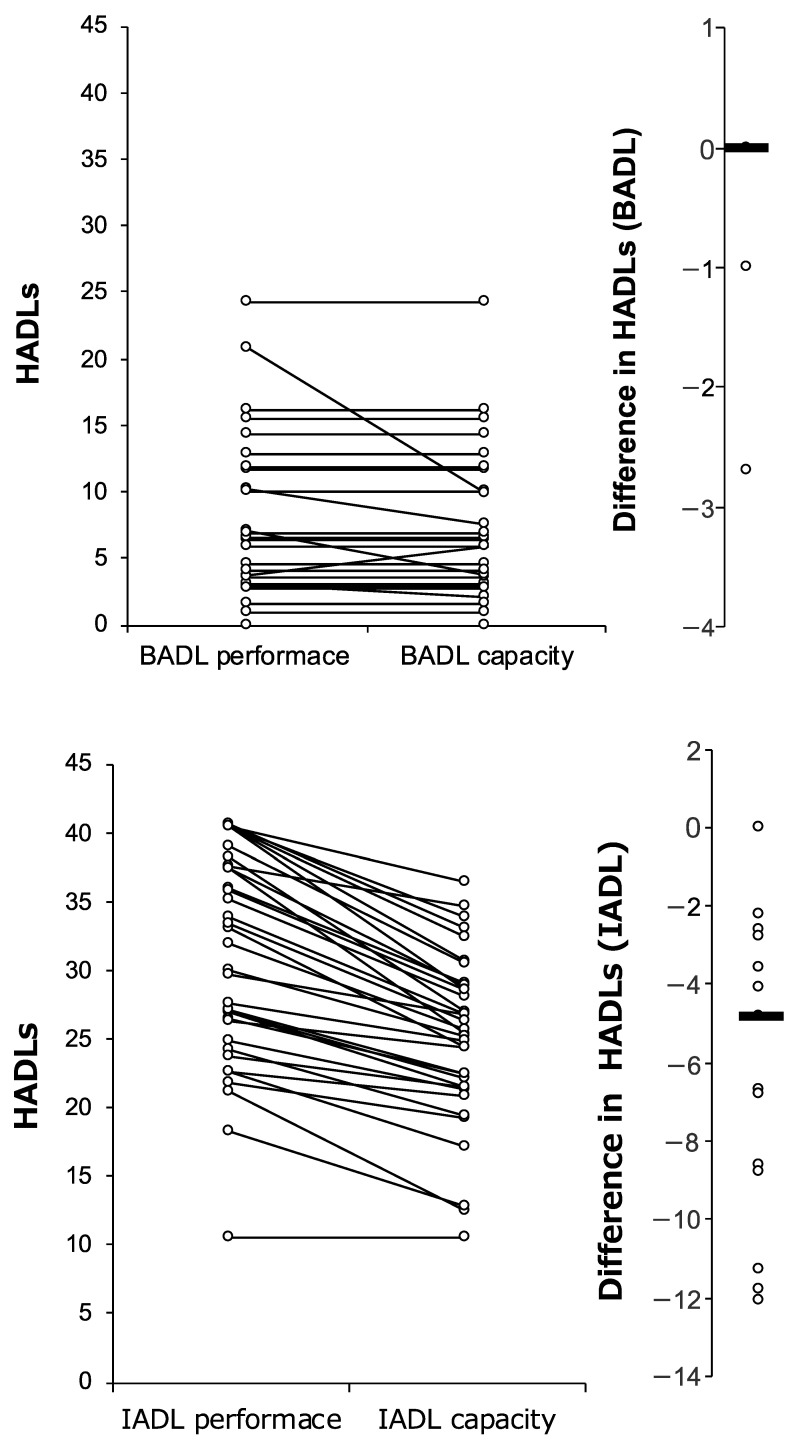
Comparisons of ADL performance versus capability for BADL and IADL as rated using the HADLS. Higher HADLS values signify lower levels of independence. The black line shows the change in performance and capacity of ADL for the same participant. The second vertical axis of the graph represented the distribution of the difference between ADL performance and ADL capacity. The bold line represents the median.

BADL performance and BADL capacity were not significantly different (*p* = 0.128); however, the participants rated IADL capacity significantly higher than IADL performance (*p* < 0.01) (Figure 1).

ADL measures were also compared separately based on the participants’ CDR status. In participants with a CDR score of 0.5, there was no significant difference between BADL performance and BADL capacity (*p* = 0.109); however, IADL capacity was rated significantly higher than performance (*p* < 0.01). Similarly, BADL performance and BADL capacity were not significantly different in participants with a CDR score of 1.0 (*p* = 0.715); however, IADL capacity was rated significantly higher than IADL performance (*p* < 0.01) (Table 1). Participants with a CDR score of 0.5 and 1.0 were significantly indistinguishable in terms of BADL performance and BADL capacity (*p* = 0.424, 0.164), IADL performance and IADL capacity (*p* = 0.187, 0.404), and total ADL performance and total ADL capacity (*p* = 0.424, 0.164).

## 4. Discussion

In this study, we investigated ADL performance and capacity in patients with mild dementia. There was no gap between BADL performance and BADL capacity; however, the scores indicated that participants performed IADL less independently than their latent potential. Dementia is a progressive chronic disease that is intractable to significant improvements in mental and physical functioning. Environmental factors are one of the contextual factors in the International Classification of Functioning, Disability, and Health (ICF) [17]. These factors make up products and the technological, social, and attitudinal environments and are important aspects that affect all components of functioning and disability [17]. Capacity reflects an individual’s ability to adjust to the environment, and the gap between performance and capacity reflects the difference between the current environment and the impact of a uniform environment [17]. Therefore, it is important to consider what changes can be made to an individual’s environment to improve their ADL performance. Our findings suggest that environmental adjustments targeting IADL are essential components of ADL-related interventions for persons with mild dementia. To the best of our knowledge, this study is the first to explicitly demonstrate a gap between IADL performance and IADL capacity in this participant population.

There was no significant difference between the participants’ actual and latent abilities to perform BADL. In the mild dementia stage, BADL are less likely to decline in the first stage. Therefore, a gap between capacity and performance is considered unlikely to occur in BADL. On the other hand, IADL, which begin to decline from the mild dementia stage, are thought to have a gap due to environmental limitations [10,11]. One could surmise from these findings that environmental adjustment is not a particularly useful strategy to improve BADL. However, there was a gap between the participants’ actual and latent abilities to perform IADL. Conventional wisdom holds that ADL decline first manifests in IADL function in people with mild dementia [10,11], suggesting that capacity assessments by caregivers in this dimension could help develop suitable care strategies [18]. In addition, older adults may not be provided with a familiar home environment [19]. The same may be true for people with dementia, and adjusting the appropriate environment may improve their performance and QOL [20]. The reported links between ADL performance status and QOL in people with dementia highlight the importance of fostering performance improvements through appropriate interventions [3,4,5]. Gitlin et al. [18,21] reported that home environmental interventions performed by family caregivers, occupational therapists, and physical therapists could effectively improve IADL independence of people with dementia and older adults living at home. Exploring interventions that can close the gap between IADL performance and capacity in people with dementia is crucial for dementia rehabilitation strategies aimed at improving QOL.

One notable point of this study is our successful identification of the gap between IADL performance and IADL capacity in persons with mild dementia by distinguishing between these concepts during the assessment. In addition, this finding supports Branch et al.’s assertions [12] that both ADL performance and capacity can offer pertinent information when examining individuals with dementia.

## 5. Limitations

This study has certain limitations and unresolved issues that require further research. First, the study area was limited. The study covered day care centers in four regions of Japan. As a future developmental task, we would like to increase the number of participating regions to cover a wide range of cities with small to large populations to avoid geographical bias, and conduct the survey with greater statistical precision. Moreover, since the study focused on mild dementia, it cannot be generalized to people with moderate-to-severe dementia. Second, the findings cannot be generalized to all persons in residential areas because we excluded individuals living alone as control variables. Third, only a few participants qualified for the study. In the future, based on the findings of this study, it may be necessary to ensure a suitable number of participants, take into account the study area and living environment, and generalize the results to persons with dementia and their caregivers.

## 6. Conclusions

Since people with mild dementia are in a situation where they are not able to fully demonstrate IADL capacity, there is ample room for intervention to improve their current situation. It is important for caregivers to make appropriate environmental adjustments in community life as these environmental interventions can contribute to improving the QOL of people with dementia.

Since no curative treatment for dementia has yet been established, it is important to improve QOL and prevent progression from an early-stage dementia, and we hope that this study will contribute to effective non-pharmacological interventions.

## Figures and Tables

**Table 1:** Demographic of the subjects and differences in performance and capacity between CDR 0.5 and 1.

	CDR 0.5	CDR 1
Gender (Male/Female)	4/8	4/19
Age (mean ± SD)	86.7 ± 5.9	86.0 ± 7.7
MMSE (median (IQR))	21 (20–24)	21 (18–22)
BADL-performance (median (IQR))	3.6 (2.6–8.2)	5.9 (3.1–10.2)
BADL-capacity (median (IQR))	3.6 (2.6–7.3)	5.9 (3.0–10.1)
IADL-performance (median (IQR))	30.1 (21.7–37.7)	31.9 (26.7–36.7)
IADL-capacity (median (IQR))	23.5 (17.6–27.8)	26.3 (21.8–28.7)

IQR: Interquartile range.

## Data Availability

The data presented in this study are available on request from the corresponding author. The data are not publicly available due to privacy concerns.
